# Tranilast, an analogue of tryptophan catabolites, induces allograft tolerance by CD161^+^ cells

**DOI:** 10.1186/1479-5876-9-S2-P27

**Published:** 2011-11-23

**Authors:** Séverine Ménoret, Séverine Bézie, Xian-Liang Li, Claire Usal, Lise Caron, Ignacio Anegon

**Affiliations:** 1 Plateform Transgenic Rats Nantes IBiSA-CNRS, Nantes, France; 2 INSERM UMR 643, Nantes, France

## Background

Indoleamine 2, 3-dioxygenase (IDO) converts tryptophan in various catabolites and has been shown to induce immune tolerance in different immune-mediated diseases, including organ transplantation. One of these tolerogenic metabolites is anthranilic acid. Tranilast is a clinically approved, structural and functional analogue of anthranilic acid that has been recently shown to be effective in murine models of multiple sclerosis and rheumatoid arthritis. We examined the effect of tranilast in a rat cardiac allograft model.

## Materials and methods

Lewis 1W rat hearts were grafted in MHC-mismatched Lewis 1A rats. The receiver is orally treated with 650mg/kg of tranilast daily for 30 days. Total splenocytes and purified spleen cell subtypes sorted by FACS Aria were transferred to sublethaly irradiated rats by *i.v* injection the day before transplantation.

## Results

Graft survival in recipients treated with tranilast were significantly prolonged (66.3±46.7 days, n=53, p<0,0001) when compared to control group (8.3±2.3 days, n=6) and in 45% of recipients (n=53) tranilast induced tolerance (>100 days survival). Adoptive transfer of total splenocytes from tolerant tranilast-treated rats to naïve rats resulted in tolerance in all animals (n=5). Moreover, splenocytes from these adoptively transferred tolerant recipients were again capable of transferring tolerance to all naïve recipients (n=5). Tolerant splenocytes depleted of T and B cells (n=6) or depleted of T, B and DCs (n=5) transferred tolerance. Importantly, depletion of CD161^+^ cells from T, B and DCs-depleted splenocytes abrogated tolerance transfer (9.3 ± 0.6 n=3). To confirm these results, we adoptively transferred CD161^+^TCR^-^ cells from tolerant rats which resulted in tolerance (130±91.6 days, n=5, 3/5 recipients >100 days) whereas CD161^-^TCR^-^ cells from the same animals did not (12.7±4.6 days, n=3, p<0,005).

## Discussion

This is the first demonstration that tranilast mediates transplantation tolerance. Tolerance was active and transferable by CD161+TCR- cells that comprise NK cells (CD161^high^) and myeloid-derived suppressor cells (CD161^low^). Experiments are under way to define which of these cell populations mediate tranilast-induced tolerance and by which downstream mechanisms.

